# Motivation Matters: Lessons for REDD+ Participatory Measurement, Reporting and Verification from Three Decades of Child Health Participatory Monitoring in Indonesia

**DOI:** 10.1371/journal.pone.0159480

**Published:** 2016-11-02

**Authors:** Dian Ekowati, Carola Hofstee, Andhika Vega Praputra, Douglas Sheil

**Affiliations:** 1 Center for International Forestry Research, Bogor, Indonesia; 2 Department of Ecology and Natural Resource Management, Norwegian University of Life Sciences, Norway and Center for International Forestry Research, Bogor, Indonesia; Wageningen University, INDONESIA

## Abstract

Participatory Measurement, Reporting and Verification (PMRV), in the context of reducing emissions from deforestation and forest degradation with its co-benefits (REDD+) requires sustained monitoring and reporting by community members. This requirement appears challenging and has yet to be achieved. Other successful, long established, community self-monitoring and reporting systems may provide valuable lessons. The Indonesian integrated village healthcare program (Posyandu) was initiated in the 1980s and still provides effective and successful participatory measurement and reporting of child health status across the diverse, and often remote, communities of Indonesia. Posyandu activities focus on the growth and development of children under the age of five by recording their height and weight and reporting these monthly to the Ministry of Health. Here we focus on the local Posyandu personnel (kaders) and their motivations and incentives for contributing. While Posyandu and REDD+ measurement and reporting activities differ, there are sufficient commonalities to draw useful lessons. We find that the Posyandu kaders are motivated by their interests in health care, by their belief that it benefits the community, and by encouragement by local leaders. Recognition from the community, status within the system, training opportunities, competition among communities, and small payments provide incentives to sustain participation. We examine these lessons in the context of REDD+.

## 1. Introduction

Reducing Emissions from Deforestation and Forest Degradation (REDD) is an international program that provides financial incentives for reducing the emission of greenhouse gases from forest lands [1–3]. Many countries have committed to related ‘REDD+’ schemes which include attention to the enhancement of forest carbon stocks, and the safeguarding of biological conservation values and local livelihoods and welfare [1]. Payments under such schemes depend on the emissions avoided as well as the fulfilment of the other environmental and social criteria. To be operational, outcome data need to be sufficiently credible (accurate and verifiable) to permit payment [4]. There is thus a need for cost-effective ways to Measure, Report and Verify (MRV) carbon stocks and other REDD+ outcomes [4].

Community participation offers opportunities for the measuring, reporting and verifying required under REDD+. Potential benefits include enhanced community engagement, opportunities for sustainability, insights into local drivers of forest cover change and on-the-ground awareness of the social and environmental factors required to safeguard co-benefits [5–8]. The Indonesian government has indicated its wish to involve local communities in such processes in REDD+ [9], though plans for implementation have yet to be developed [10].

Developing MRV systems in a vast and diverse country like Indonesia, poses challenges. Indonesia is an archipelago of 17,500 islands, with a culturally diverse population of more than 250 million, spread over 1.9 million km^2^ often with difficulties of access and communication, where trained professionals are typically scarce or absent over large areas [11]. Clearly, any information or insight on how such systems might be designed and implemented to function effectively would be valuable.

REDD+ remains a comparatively new concept. Most REDD+ and associated Participatory Measurement, Reporting and Verification (PMRV) experiences are short term and at limited spatial scales [8,12,13]. Thus we lack examples of durable and large-scale PMRV for REDD+. This apparent dearth of examples helps fuel the scepticism that such schemes could ever be operational [14]. Experiences from small scale pilot activities have underlined the importance of engaging with the right people, ensuring they possess suitable skills, and maintaining their motivation [8]. At larger and longer-term scale we lack guidance derived from REDD+ projects. Fortunately, useful insights can be found elsewhere.

Angelsen [15] highlighted “The challenges that arose in other sectors are highly relevant to REDD+, but the lessons are rarely brought into the REDD+ debate. They should.” A sector he took as example in his paper was health. Little research has yet addressed this. Angelsen’s own tentative assessments focused on REDD+ and ‘performance based’ aid, see [15]: he highlighted the apparently positive role such aid had played in the health sector though the evidence was often anecdotal (citing the review by Eldridge and Palmer 2009 [16]) and Öhler et al. 2012 in governance reform [17].

Another example is study on how REDD+ might address corruption by noting lessons from other sectors [18]. We found no examples focused on sustained national monitoring and reporting. Here we address this gap by examining the success of Indonesia’s successful village healthcare “Posyandu” program.

The Indonesian integrated village healthcare program (Posyandu) demonstrates that data collection by community members can be successfully implemented and maintained across the nation’s diverse lands and communities. The Posyandu program was established, as part of the national child and maternal health program in the 1980s. Its subsequent achievements include improved nutritional status of children [19] and substantial reductions in the mortality of children under the age of five [20] from 119 per 1000 live births in 1980 [21] to just 29 in 2013 [22]. The number of Posyandu has grown from around 25,000 in 1985, and 244,000 in 1990 [23], to 280,000 across the nation in 2013 [24].

While Posyandu and REDD+ have different goals; the scale and ambition of the reporting efforts and associated challenges are similar. Even the goals may be more similar than is initially apparent given that healthy children and healthy environments are near universally valued even if there is considerable variation in emphasis [25–29]. Furthermore, views on what defines and determines healthy children and healthy environments are not fixed but can be influenced, see *e*.*g*. [30]. The measurements and observations required for effective monitoring, be it the height and weight of children or the stem-diameters and densities of a forest are tangible and close to daily life. In both cases, the benefits from monitoring relate to people, their families and their communities.

Fundamentally, efforts towards improved health and environmental outcomes are subject to human agency within a wider social and institutional context. Theories of individual and collective agency apply. In these theories motivations are not restricted to financial transactions and incentives, but include pride, beliefs and other factors [31,32]. Thus an individuals’ choices and performance may depend on how they value an activity and its outcomes, and how their own contribution is perceived [33]. Whether monitoring child health or environmental outcomes, the level of community engagement depends on perceptions, motives and incentives.

We see growing interest on how motives and incentives influence participation. In psychology and economics, there recognition that intrinsic motivation is often key [34–36]. Personal motivations explain involvement in, and success of, volunteer-based biodiversity monitoring in six European countries [37] and community-centred ecosystem monitoring in Canada [38]. In the context of REDD+, a study suggest that community perceptions of relevance (e.g. perceptions of forest benefits and understanding their role in MRV) will determine engagement with MRV [34].

Motivation and incentives remain little studied in the context of environmental assessments and reporting from tropical forest countries. Incentives are a recognised ‘information gap’ in participatory monitoring [39]. We know that community members in many remote communities effectively police access to their territories through personal and community level processes of observations and enforcement [40]–indicating that the motivations within such communities are sufficient for the task in these cases. Other studies, looking more specifically at PMRV, have also suggested that motivation and incentives affect participation [11] and its sustainability [34].

Here we ask what PMRV for REDD+ can learn from the Posyandu program. We examined how data collected by village level personnel (‘kader’) is gathered and flows through a multilevel system to reach the national database. We explored the motivation of the kader, their access to incentives, and their views about the system.

## 2. Methods

### 2.1. Research sites

**Fig 1:**
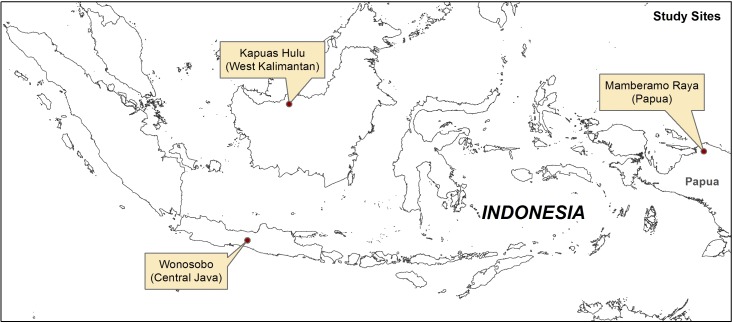
Research sites in Papua, West Kalimantan and Central Java.

This study was conducted in seven villages, in three districts in Indonesia: Wonosobo, Kapuas Hulu and Mamberamo Raya in the three provinces of Central Java, West Kalimantan and Papua respectively (Fig 1). The three provinces differ in their autonomy [34], in their population density and the number of Posyandu (Table 1). Since decentralization began in 1999, the districts have been enjoying increased autonomy, including some control over budgets. Due to their ‘special autonomy’ status, districts in Papua have the most freedom in budget (including the health budget) management [41,42]. This study contributes to a larger multidisciplinary project examining the feasibility of PMRV for REDD+ in Indonesia in the same locations [11]. The communities had not hosted or been impacted by past or present REDD+ projects or anything similar that might have influenced their views [11].

**Table 1:** General information on our study districts in Papua, West Kalimantan and Central Java.

District	Population	Population density/km^2^	Number of Posyandu	Ratio of Population per Posyandu
**Mamberamo Raya**	12,300	0.4	4	3075
**Kapuas Hulu**	244,000	8	300	813
**Wonosobo**	758,000	770	1246	608

**Data sources:** Population and Posyandu numbers are from Village Potential Statistics 2011 [43]. Population densities were derived from the total population divided by the district area from data obtained from: Mamberamo Raya District Health Service [44], Pemerintah Kabupaten Kapuas Hulu [45], and Pemerintah Kabupaten Wonosobo [46]

The Posyandu in the three provinces vary in the number, accessibility and infrastructure. Comparing the number of Posyandu and population, a Posyandu in Kapuas Hulu or Wonosobo is for less than one thousand people, while a Posyandu in Mamberamo Raya is for more than three thousand (Table 1). Wonosobo District in Central Java has well-established roads, good mobile phone and internet connection, and is therefore more accessible than the other two sites. Kapuas Hulu District in West Kalimantan has a medium level of infrastructure and villages can be accessed by road, but during the rainy season some villages are inaccessible to motorized vehicles. Telecommunication signals are sporadic and patchy in some villages. Mamberamo Raya District in Papua is a remote area with challenging transportation where boats are often the only transport. Challenges include infrequent availability and high fuel prices, and limited telecommunications though many villages have short band radios.

### 2.2. Posyandu: what are they and how do they compare to PMRV requirements?

The main objective of the Posyandu when it was established in the 1980s, was to decrease the mortality rate of children under the age of five by closely monitoring growth, identifying stunted growth (a primary manifestation of malnutrition) and treating its underlying causes.

To achieve this objective, the Ministry of Health at the provincial and district levels require regular health data from the villages to develop, implement and adjust nutritional and medical programs. Therefore, Posyandu kaders recruited at the village level measure and weigh children each month and report these result up the hierarchy. The data flows from the kader up through the sub-district, district and provincial levels until it reaches the national level (the Ministry of Health). We note that this institutional model is similar to what seems likely for PMRV in REDD+, where data are expected to flow from the village level through sub-district, district and provincial levels until it reaches a designated institution at the national level.

The Ministry of Health published and distributed guidelines for Posyandu implementation throughout Indonesia in 2011 [47]. These guidelines define a kader as a community member with the capacity, time and willingness to work and who is selected for Posyandu duties. The guidelines do not clarify any particular ‘capacity’ requirements. From our reading of the guidelines they suggest that the best available candidates be selected, and after selection be given some training [47]. Only 5–15 people from each village become active kader. Again we see parallels with PMRV for REDD+, where only selected people are likely to be involved and trained.

### 2.3. Informant selection

We conducted 70 interviews in total. We focused our attention on informants at the village level, but included sub-district, district, provincial and national levels (Table 2). All informants were involved in monitoring, reporting and validation of growth data on children under the age of five.

**Table 2:** Number of informants and types of interview conducted by district.

Level of informants	Informants		Papua	West Kalimantan	Central Java	Total
**Village**	Posyandu kader	Individual interviews	7	3	8	18
		Group interviews	0	5	1	6
		Total interviews	7	8	9	24
		Total informants	7	22	10	39
**Village**	Village midwife/ village nurse (a staff of sub-district healthcare clinic who is assigned at each village)	Individual interviews	2	2	2	6
		Group interviews	0	1	0	1
		Total interviews	2	3	2	7
		Total informants	2	4	2	8
**Sub-district**	Nutrition/maternal and child health section staff in sub-district healthcare clinics	Individual interviews	2	1	2	5
		Group interviews	1	1	1	3
		Total interviews	3	2	3	8
		Total informants	4	3	4	11
**District**	Nutrition/maternal and child health section staff in the district health service	Individual interviews	2	1	1	4
		Group interviews	1	0	1	2
		Total interviews	3	1	2	6
		Total informants	4	1	3	8
**Province**	Nutrition/maternal and child health section staff in provincial health service	Individual interviews	1	1	1	3
		Group interviews	0	0	0	0
		Total interviews	1	1	1	3
		Total informants	1	1	1	3
**National**	Directorate general for nutrition staff in the Ministry of Health	Individual interviews	Not applicable			1
		Group interviews	Not applicable			0
		Total interviews	Not applicable			1
		Total informants	Not applicable			1
**Total interviews at all levels**			16	15	17	**49**
**Total informants at all levels**			18	31	20	**70**

### 2.4. Interviews

We used open-ended questionnaires. We asked informants about the kaders’ motivation, incentives, factors which sustain the system and recommendations to improve the Posyandu. We define ‘motivations’ as factors internal and ‘incentives’ as factors external to the actor involved. Only informants at the village, sub-district, and district level were asked about the kaders’ motivation. We asked informants at all levels questions on incentives, system sustainability and effectiveness, as well as strengths, weaknesses and recommendations.

### 2.5. Ethic statement

This research on ‘Motivation matters: Lessons for REDD+ participatory measurement, reporting and verification from three decades of child health participatory monitoring in Indonesia’, including the ethics procedures, was approved by the Center for International Forestry Research (CIFOR) and the Government of the Republic of Indonesia. This included all the authorizations required to conduct this research. CIFOR does not have a specific ethics committee but senior management reviews all CIFOR research prior to execution. Specifically, our study was approved by CIFOR before we applied for and obtained a research permit and travel permits from the Ministry of Home Affairs based on the proposal. As for the Ministry of Health, no separate ethical approval was sought since we did not seek or require personal healthcare data on either adults or children as a part of the study. We obtained verbal consent from our informants. We did not obtain written consent as this was neither necessary nor practical as, although most kader can write, overall literacy levels were low and written statements lack validity in such a context.

The informants were identified in several ways but mostly by snowball sampling from a lower or higher level of governance, partially due to the high rates of rotation within the Ministry of Health (MoH). At the very beginning, we contacted the district health office in Bogor to determine which informants and divisions within the MoH could be approached for information. In Papua we started at the provincial level, we identified the informant through referral down the network depending on where we started. After obtaining contact details, we arranged an interview. In Central-Java and West Kalimantan this was reversed: we started at the village level and later asked who they reported to and contacted that person requesting an interview.

Before starting the interview, we explained our goals and asked if the informant had any questions regarding our activity. We always left a copy of our research proposal (in Indonesian) and our contact details with each informant. We asked the informants to contact us at any time if they had any questions and/or concerns. Wherever possible, we interviewed our informants individually. However, some informants, mostly at the village level, indicated a strong preference to be interviewed as a group and we accommodated this request (see Table 2).

### 2.6. Analysis

We categorized the informants’ answers as follows: Posyandu success, motivation, financial incentives, factors perceived as important to sustain its operations and recommendations to improve efficiency. We grouped similar responses and used these groupings to code each answer as categories (see results). We compared the occurrence and frequency of these coded responses. While these data are qualitative in nature we wish to highlight the difference between widespread versus individual concerns and their distribution.

## 3. Results

### 3.1. Posyandu success

Indonesia’s Posyandu system provides an example of a successful, long-lasting program initiated by the government and supported by local communities. All informants in all the sites supported the Posyandu and its continuation; despite recognising various challenges views were positive and constructive overall. Indeed, the informants were keen to suggest ways and means to improve Posyandu effectiveness. For example, in Papua where mobile phone coverage was limited, our informants at the district level, proposed each village should receive a Single-Sideband Radio (SSB radio) to ease communication problems and improve their response to health emergencies.

Several informants indicated that the Posyandu had not always had popular support, but had gradually won the backing of the community through its achievements (personal communication with village and sub-district level informants in Central Java). In West Kalimantan, villagers expressed their pride in the Posyandu and noted that it would be a shame to lose it–especially if other villages still had theirs. In Papua, despite various grievances with government agencies and kaders in their village, villagers expressed their need for the Posyandu to maintain their children’s health (personal communication with village informants).

The success of the Posyandu in maintaining its kader is shown by the long service of some kader. The service of our kader informants averaged 2.8 years in West Kalimantan, 3.1 years in Papua and 10.1 years in Central Java. The range (in years) for each site was 1–6, 1–9 and 1–31 respectively.

The capacity of the kader has also contributed to the success of the Posyandu. Informants noted that the required ‘capacity’ was not well defined by the Ministry of Health and varied with location. In West Kalimantan and Central Java, the kader appear to be selected primarily for their literacy, while in Papua the kader are selected primarily for their background and experience with maternal and child health care issues. In Papua, non-literate kader were assigned tasks such as preparing supplementary food, helping to calm the children during the Posyandu activities and cleaning the premises where reading and writing were unecessary.

Six informants from Papua, 11 from West Kalimantan, and 1 from Central Java, out of 18, 31, 20 informants respectively stated that one way to improve the kaders’ capacity was to provide training. We found from our interviews that kader training seldom involved any formal teaching from government healthcare staff, but generally included on the job guidance from senior kader. Most kader stated that for most of the basic work, such as weighing and recording, this training was sufficient.

### 3.2. Response categories

We categorized the responses to the various questions to clarify shared themes. Under factors contributing to Posyandu success our response categories include ‘the gradual acceptance of Posyandu by the community’, ‘the length of kaders’ service’, ‘capacity’, and ‘ways to improve capacity’ codes. Suggestions provided as motivation were categorized as ‘wish to benefit the community’, ‘personal interest in childcare’, ‘engaged by respected person’, ‘feeling of responsibility’, ‘opportunity to learn’, and ‘availability of spare time’. For financial incentives, we had only two categories ‘amount’ and ‘transparency’. Suggested factors perceived as important to sustain Posyandu operations were categorized as ‘motivated kaders’, ‘community support’, ‘financial incentive’ and ‘government regulation’. Under recommendations to improve Posyandu’s efficiency, our categories were ‘increase support from upper levels of Posyandu (financial and/or non-financial)’, ‘financial incentive/funding for Posyandu’, ‘training for kader’, and ‘improving Posyandu facilities’.

### 3.3 Kader motivation to participate in Posyandu activities

All informants indicated that the motivation to join the Posyandu reflected the benefits for the community and the kaders’ personal interest in childcare activities (Table 3). Such personal interest was revealed by previous involvement in childcare activities, for example. Six informants from Papua, 8 from West Kalimantan, and 6 from Central Java, out of 18, 31, 20 informants respectively noted that they had been personally asked to become a kader by a respected person. In Central Java, religious values were noted as a strong motivation to join though this was not found in other sites. In West Kalimantan (9 informants) and Central Java (1 informant) implied that they do it because no one else wanted to. Only in West Kalimantan was village pride a stated motivation. Some kader said they would be ashamed if they did not have a Posyandu while their neighbouring villages did or if their Posyandu had to be operated by kader from neighbouring villages. When we asked the village nurses and midwives (staff of sub-district healthcare clinic who are assigned at each village), healthcare staff at the public healthcare clinics and district health services about the motivation of the villagers to become kader, all informants at all the sites indicated that the wish to benefit the community was the dominant motivator followed by having a personal interest in the work and its goals.

**Table 3:** What motivates villagers to become Posyandu kader (58 informants from the village, sub-district and district levels).

		Wish to benefit the community	Personal interest in childcare	Engaged by respected person	Feeling of responsibility	Opportunity to learn	Availability of spare time	Other answers
**Kader**	Papua	✓	✓	✓		✓		
	West Kalimantan	✓	✓	✓	✓	✓	✓	• Village pride • Easy to do
	Central Java	✓	✓	✓	✓		✓	• Support from spouse • Looking for God’s reward in heaven
**Village midwife/ nurse**	Papua	✓	✓					
	West Kalimantan	✓						
	Central Java	✓						Self-fulfilment
**Sub-district healthcare clinic**	Papua		✓					
	West Kalimantan	✓		✓				
	Central Java	✓			✓			
**District health service**	Papua	✓	✓		✓			
	West Kalimantan	✓						
	Central Java	✓						

### 3.4. Financial incentive kader receive for participating in the Posyandu

Levels of Posyandu participation by kader were relatively similar among sites. Formal Posyandu tasks undertaken by the kader were generally restricted to just one day per month in all the sites studied. Sometimes the kader spent additional time to visit families that would otherwise not be included under Posyandu.

All kader receive a payment for their work (Table 4). This payment is called ‘transportation allowance’ (uang transportasi) in West Kalimantan and Central Java and ‘salary’ (gaji) in Papua. In 2014, Papuan kader received by far the largest sums (around 26 USD per month). Kader in West Kalimantan received USD 2 and Central Java received less than USD 1 per month and they agreed that the amount was far below the average daily labour rate.

**Table 4:** Budget availability for Posyandu kader incentives (70 informants from village-national levels).

	Papua	West Kalimantan	Central Java
**Amount of salary**	• Kader receive IDR 300,000 (+/-USD 26) per month • Paid every 3 months.	• Kader receive IDR 25,000 (+/-USD 2) per month • Paid monthly.	• Kader receive IDR 5,000 (+/-USD 0.5) or IDR 10,000 (+/- USD 0.9) per month • Paid annually.
**Source of fund**	• District government through district health service	• Ministry of Health (health allocation fund via health services) • Ministry of Home Affairs (village allocation fund via village government)	• Ministry of Health (health allocation fund via health services) • Ministry of Home Affairs (village allocation fund via village government)

Informants had different perceptions of financial transparency depending on their province. In West Kalimantan, there were two sources of funds for Posyandu (see Table 4). No issues were raised by the kader about the allocation of health specific funds distributed by the sub-district health center clinic. However, they indicated that their village government was not transparent in distributing other Posyandu funds as part of the village budget. The sub-district healthcare clinic and the district services informants in West Kalimantan stated that they could not solve the problem because of its cross-sectoral nature. Central Java has a similar source of funds to West Kalimantan, but there were no concerns about transparency. Papua informants indicated that there was one source of funds for Posyandu, i.e. a health specific fund distributed via the district health service, which they believed was transparent.

An informant from the Ministry of Health (national level) stated that the distribution of the health allocation fund was challenging due to the inability of the district service staff to manage the fund and produce accountable, financial reports and that these challenges have increased since decentralization in 1999. Administrative staff tend to be moved around every five years or so (whenever a new district head is instated) which disrupts continuity and reduces the experience and knowledge of managers.

### 3.5. Factors perceived as important to sustain Posyandu operations

Most informants stated that active and motivated kader, village midwives and nurses are important in sustaining the Posyandu (Table 5). Community support, as well as support from the health government staff, and the higher governance levels, also contribute. However, our village midwife/nurse informants in West Kalimantan and Central Java noted a decline in community support and therefore a decrease in kader motivation. This is indicated by the decrease in the number of Posyandu visitors. The two most common reasons offered were the proximity of competing low-cost health clinics (Central Java) and that families are busy with their gardens or farms (West Kalimantan). The fact that some farming families reported being “too busy with their gardens”, suggests a decline in support for the Posyandu.

**Table 5:** Perception of 70 informants of important factors that sustain the Posyandu program (informants from village-national levels).

		Active kader	Active village midwife/nurse	Support from healthcare government staff (including midwife/nurse), village government	Community support	Reward for kader (salary, training, etc.)	Initiated by central gov’t/obliged	Funding for Posyandu general operation
**Kader**	Papua	✓	✓		✓			✓
	West Kalimantan	✓	✓		✓			
	Central Java		✓	✓	✓		✓	
**Village midwife/ nurse**	Papua					✓		✓
	West Kalimantan	✓	✓					
	Central Java	✓		✓	✓			
**Sub-district healthcare clinic**	Papua		✓					
	West Kalimantan	✓		✓	✓			
	Central Java	✓		✓		✓	✓	
**District health service**	Papua	✓				✓		
	West Kalimantan				✓		✓	
	Central Java	✓						
**Provincial health service**	Papua	✓						
	West Kalimantan			✓		✓		
	Central Java			✓				

Only three informants (a village nurse and a district health service staff in Papua, and a sub-district healthcare clinic staff in Central Java, no kader) explicitly mentioned their belief that financial incentives played a major role in motivating kader. In West Kalimantan two kader stated that the kader’s sense of belonging to the Posyandu helped ensure Posyandu success.

An informant from the Ministry of Health (national level) said that regulation No. 38/2007 sustains Posyandu. This regulation states that the Ministry of Health has the authority to replace the district head if districts fail to report their nutrition data (as derived primarily from Posyandu activities and reporting).

### 3.6. Informants’ recommendations to improve Posyandu efficiency

When we asked informants about recommendations to improve the program’s efficiency, 9 informants from Papua, 27 from West Kalimantan, and 5 from Central Java out of 18, 31, 20 informants respectively highlighted the benefit of increased support (Table 6). The informants also agreed that the kader deserve rewards for their work and achievements. Many of them specifically proposed either an increase in financial incentives for the kader or more funding for the program. Other suggestions included more training, supervision and higher-level feedback.

**Table 6:** Recommendations from 70 informants to improve the Posyandu program (informants from village-national levels).

		Increase support from upper levels of Posyandu* (financial and/or non-financial)	Financial incentive/ funding for Posyandu	Training for kader	Improving Posyandu facilities	Additional note
**Kader**	Papua	✓		✓		Posyandu should be more autonomous and separate from the village nurse
	West Kalimantan	✓	✓	✓	✓	
	Central Java	✓	✓		✓	
**Village midwife/nurse**	Papua		✓		✓	
	West Kalimantan		✓	✓	✓	
	Central Java		✓	✓	✓	
**Sub-district healthcare clinic**	Papua	✓		✓		
	West Kalimantan		✓	✓		
	Central Java	✓				
**District health service**	Papua	✓			✓	More HCWs
	West Kalimantan	✓				- Collaborate with other sectors (private) - Research
	Central Java	✓	✓			
**Provincial health service**	Papua					Revitalization
	West Kalimantan	✓				
	Central Java					Increase HCW capacity

*Upper levels of Posyandu refers to village healthcare staff and sub-district up to national level health service staff

Our national level informant emphasized the need for closer collaboration between all governance levels to achieve and maintain regular and timely reporting. This informant emphasized that this collaboration had become more necessary following decentralization. Before decentralization, the informant said, the Ministry of Health could more easily request a report from the health services in the provinces and districts. Today, they often experience extended delays as the requests and reports pass through the multiple levels of government.

## 4. Discussion

Indonesia is a vast, diverse and logistically complex country. The remarkable success of the Posyandu appraoch shows that–in the context of health care at least–a participatory monitoring and reporting system can work and be sustained in such a context. Despite many challenges, effective community monitoring and reporting in Posyandu has been maintained for three decades [10, 11]. Community support was gained, and as we have shown, many kader have remained committed to the process for many years. This does not prove that PMRV for REDD+ will necessarily succeed, but it can indicate ingredients that might contribute to success. So besides providing inspiration, what can the Posyandu tell us about ensuring successful measurement, reporting and verification systems in the context of REDD+?

### 4.1. Choosing the right people

The right people are those who believe in the activity, and the benefits that their contribution can provide. In the case of Posyandu this means benefits for their community, their family and themselves. Such individuals are likely to have a personal interest in childcare and a belief that such activities are worthy and respectable. Another large-scale study of Posyandu, found that villagers who have young children are more likely to contribute actively to the Posyandu compared to villagers without young children [48]. Our observations show that motivations are more diverse and often unselfish. For example, not all kader have young children themselves, though all appeared motivated by concern for good childcare. This suggests that REDD+ PMRV is more likely to succeed if villagers are concerned by environmental values, forest protection, and other social and developmental goals recognised under REDD+. Developing such aspirations would also bolster support for REDD+ thus making its goals easier to achieve.

The enduring nature of kader involvement, along with the various views and how they are expressed, indicates pride and ownership within the kaders. They express it even when they say ‘our Posyandu’, despite it being a government program. The informant in Central Java who has been a kader for 31 years said she did not believe the newer kader had the skills and sufficient experience to be left in charge and so she has not yet retired. Kaders in West Kalimantan that joined Posyandu for 6 years highlighted their personal pride in ensuring their Posyandu’s continuing effectiveness. The Kader in Papua, who have been with the Posyandu for 4–9 years, continue to run their Posyandu even when the village nurse is absent.

Such pride and ownership can be encouraged. The broader health education role of the Posyandu was considered weak in the past and has subsequently been strengthened [49]. Posyandu began as an outsider led activity, but we can see that once the value of the Posyandu system became apparent it gained local appreciation and respect. This confirms other studies’ findings that voluntary activities and support for communal benefits can be generated by outside agencies resulting in communal benefits [50]. While any new program may meet some initial scepticism, the successful implementation of the Posyandu approach included large-scale education on health, nutrition and family planning [23,51]. One example of this popular outreach is the promotion of a popular song titled “I’m a healthy kid” (Aku anak sehat) with lyrics that mentioned regular Posyandu visits in its lyrics. In our interviews, we found that this appreciation of the Posyandu system was still promoted by government officials and by village leaders. REDD+ is also an externally driven program that can be presented as relevant and beneficial to communities, and where investment in sensitization, education and popularization is likely to be beneficial.

Our observations of the Posyandu system highlight the role of respected leaders in selecting and motivating kader [51–53]. The community leaders’ request for villagers to join the kader ranks appears to work in two ways. *First*, the requested villagers feel socially responsible to join and hesitate to decline, and they increase their social status because of the invitation. *Second*, community leaders live there and interact daily with the community. Such leaders will likely have good insights regarding who may best be involved in any activities and who should be targeted as potential champions of REDD+.

Engaging leaders in a constructive manner to ensure their support is clearly important in both health care and environmental stewardship. In Indonesian rural communities there are typically three sources of authority (government, customary and religious) [54], while teachers and others can, of course, also play an important role. In Central Java, religious values motivated villagers to join the Posyandu suggesting that in this location collaboration with religious leaders was especially important while other authority figures dominated in other areas. Since childcare is often perceived as a mother’s issue, the role of women was shown to be vital especially in central Java and West Kalimantan Posyandus. This was less evident in Papua, where women and men appeared more equally involved in childcare. Such orientation depends on multiple factors including the disposition of the individuals (including gender preference and who might be more interested in such activities in certain sites) involved, contexts and leadership roles. Other than healthcare, we see similar experience that highlights the local leadership role in sanitation [55] and in community based fisheries [56]. The wider lesson is the necessity of a flexible approach to finding champions with the interest and ability to promote community engagement.

### 4.2. Keeping the right people motivated: incentives matter

Once engaged, what is it that keeps kader members motivated? Do incentives matter? The existing literature on incentives for REDD+ for the most part lack relevance for PMRV because they consider landholders and forest users rather than potential MRV informants [57–60]. Willingness to engage with REDD+ does not necessarily imply that people have suitability, time, skills and motivation to be involved in monitoring and reporting. Some suggest that financial incentives are necessary (Edwards *et al* 2010 and Corbera and Schroeder 2011 in [8][61]. However, it remains unclear when and to what extent financial incentives are necessary as there is very little research on this, and we know that people and communities do their own monitoring for their own reasons e.g., see [15]. Rather than being a financial compensation for time and effort, payment may play a symbolic role in indicating recognition and respect. Other motives matter. We know from research in other parts of the world that it is often possible to find people sufficiently motivated to work for next-to-nothing in difficult circumstances, e.g., palliative care volunteers in central Africa where the “wish to help people” is a key motivator along with a resulting sense of pride and status [62].

Our own findings concerning Posyandu success show that while incentives matter, they are neither solely, nor primarily, financial. Recognition and opportunities for training and career development are also important. Others have highlighted the role of recognition within their community rather than simply financial incentives in motivating kader participation in Posyandu monitoring and reporting activities [49]. When the financial incentive is small, community recognition/support for the Posyandu activities are the key reasons kader members continue their work. Our findings show that being recognized as a kader provides satisfaction and increased social status within the community. Recognition for PMRV members is something that can and should be promoted.

Besides recognition within their community, we see that recognition within the system also matters. This requires regular feedback and supervision from higher levels of the system [48,50,51]. In the context of community monitoring in REDD+, feedback is also important for maintaining participation and data quality [8]. Most of our Posyandu informants indicated that they receive regular supervision and feedback on their activities and reports from the village midwives and nurses–except for Papuan kaders. Despite this, many kader expressed a desire for more regular feedback, encouragement and recognition for their achievements. Such feedback will be essential to keeping PMRV teams engaged and effective.

We have seen how pride and competition between communities act in a positive way to motivate the communities–as seen in West Kalimantan—where informants highlighted this as a major motivation. The development of small competitive awards for good performance can help generate such motivations. Such awards could be harnessed for PMRV.

Organized training programs at the sub-district and district levels were indicated as motivations [63]. Within the environmental field, it was noted that training for the local community is often key to parataxonomists and parabiologists involvement in biodiversity studies [64–66] and to REDD+ PMRV [8] and is likely to play a significant role in maintaining such activities. In our interviews all our local informants wished for more training. As most kader spend most of their life in their village, training elsewhere, aside from its function to learn/strengthen their skills, is considered special and desirable. These appear to combine multiple factors: formal recognition and reward, career development, status, and the simple pleasure of travelling outside their community and sharing views and experiences with like-minded people. Training opportunities would help maintain interest in PMRV.

The Posyandu PMRV system has achieved an impressive record of accomplishment. Proposed REDD+ PMRV systems may one day contribute similarly to sustaining environmental health. The desire for good health and for a good environment appears universal, and both may be able to motivate community support. In this way what began as an externally driven initiative has the potential to win and sustain support. Nonetheless, while poor health is relatively easily agreed, the nature of an undesirable environment may be less universal, involving trade-offs with other desirable outcomes such as financial opportunities, and thus a clear consensus on desirable versus undesirable REDD+ related outcomes may not coincide with community perceptions [67–70]. We speculate that support for REDD+ will likely require a greater investment, at least initially, in consensus building and incentives than was required in Posyandu and its child healthcare goals.

Generating popular support for REDD+ activities will require investment in environmental awareness and the belief that REDD+ activities deserve support. While far from trivial, this aspiration is not impossible. We already see significant concern about forest loss and degradation and associated issues in even the most remote communities, e.g. in Kalimantan [71–74]. Likewise, there are examples of various communities striving to manage their natural resources sustainably, e.g. Dayak in Kalimantan, Krui in West Lampung, Meru Betiri in East Java [75], Kasepuhan in West Java [76] or Mamberamo in Papua [77,78]. Integration of environmental awareness into school lessons is already ongoing. And there is considerable literature on sharing experiences among communities through activities such as cross-site visits that allow people to see and judge for themselves [79–81]. These experiences suggest that building local support for REDD+ and PMRV is possible and would bring improved environmental awareness and other benefits.

We have not examined how community involvement can improve choice, actions and outcomes–though we suspect that benefits exist. Kaders are living their daily life in their communities, seeing their neighbours every day, regularly making observations and can identify when urgent local interventions are needed and will thus react accordingly outside of the formal reporting system. This is something that has been emphasized previously in participatory forest monitoring for collaborative management [82], but has gained less emphasis in REDD+ PMRV as currently defined. Monitoring and management have become disconnected in modern natural resource management with data collection becoming an end in itself [83–85]. Empowering community members not only as observers but active informants can address this deficiency. Such awareness and empowerment could, if handled appropriately, not only improve PMRV, but also improve outcomes [66,82,86].

## 5. Conclusion and Recommendation

Indonesia’s Posyandu indicates that sustained large-scale participatory monitoring and reporting can be achieved–at least in the health care system–in an affordable and effective way across Indonesia. Both lessons and inspiration may be drawn from this example. The lessons include the diversity of motivations and incentives that contribute to sustained participation. A sense of ownership and pride appears particularly valuable. Such motivations have sustained effective participation despite limited financial incentives and external oversight. Belief in the cause is crucial though specific details vary. In Central Java, recognition from religious leaders played a key role, in Central Kalimantan competition among communities and local pride in their achievements and in Papua training opportunities motivated the kader.

We see an urgent need to engage and motivate people to protect and enhance their environment. If REDD+ is perceived as leading to valued and desirable outcomes PMRV activities will be easier to develop, implement and sustain.

## Supporting Information

S1 Text Questionnaires. (RAR)
